# 60-Day Chronic Exposure to Low Concentrations of HgCl_2_ Impairs Sperm Quality: Hormonal Imbalance and Oxidative Stress as Potential Routes for Reproductive Dysfunction in Rats

**DOI:** 10.1371/journal.pone.0111202

**Published:** 2014-11-04

**Authors:** Caroline S. Martinez, João Guilherme D. Torres, Franck M. Peçanha, Janete A. Anselmo-Franci, Dalton V. Vassallo, Mercedes Salaices, María J. Alonso, Giulia A. Wiggers

**Affiliations:** 1 Postgraduate Program in Biochemistry, Universidade Federal do Pampa, Uruguaiana, Rio Grande do Sul, Brazil; 2 Department of Morphology, Stomatology and Physiology, School of Dentistry, Universidade de São Paulo, Ribeirão Preto, São Paulo, Brazil; 3 Department of Physiological Sciences, Universidade Federal do Espírito Santo, Vitória, Espírito Santo, Brazil; 4 Department of Pharmacology, School of Medicine, Universidad Autónoma de Madrid, Madrid, Spain; 5 Department of Biochemistry, Physiology and Molecular Genetics, Universidad Rey Juan Carlos, Alcorcón, Spain; University Hospital of Münster, Germany

## Abstract

Mercury is a toxic and bio-accumulative heavy metal of global concern. While good deals of research have been conducted on the toxic effects of mercury, little is known about the mechanisms involved in the pathogenesis of male reproductive dysfunction induced by mercury. Therefore, the purpose of this study was to assess the effects and underlying mechanisms of chronic mercury exposure at low levels on male reproductive system of rats. Three-month-old male Wistar rats were divided into two groups and treated for 60 days with saline (i.m., Control) and HgCl_2_ (i.m. 1^st^ dose: 4.6 µg/kg, subsequent doses 0.07 µg/kg/day). We analyzed sperm parameters, hormonal levels and biomarkers of oxidative stress in testis, epididymis, prostate and vas deferens. Mercury treatment decreased daily sperm production, count and motility and increased head and tail morphologic abnormalities. Moreover, mercury treatment decreased luteinizing hormone levels, increased lipid peroxidation on testis and decreased antioxidant enzymes activities (superoxide dismutase and catalase) on reproductive organs. Our data demonstrate that 60-day chronic exposure to low concentrations of HgCl_2_ impairs sperm quality and promotes hormonal imbalance. The raised oxidative stress seems to be a potential mechanism involved on male reproductive toxicity by mercury.

## Introduction

Mercury is a persistent toxic and bio-accumulative heavy metal of global concern since their anthropogenic and natural emissions still pose high risk to human and environmental health. The main contribution to human exposure is the ingestion of fish and seafood (methylmercury); however, it can also occur from dental amalgam fillings (metallic mercury), vaccines (ethylmercury) and contaminated water and air (mercury chloride) [1], [2].

Human exposure to mercury is still high even with the recommendations from international regulatory agencies. Within the US population, the chronic mercury exposure and average population of mercury concentrations rose sharply from 1999 to 2006 [3]. At Tapajós River Basin, Amazon, Brazil, known for mercury occupational exposure from gold mining and, consequently, bioaccumulation, exposure to mercury have decreased between 1999–2012 among women of reproductive age; however the potential risk is still an issue because 22% of the women shows high mercury levels. In the aforementioned cohort studies, the mercury levels are close to or above the tolerance limit established by WHO [4]–[6].

Mercury in a cumulative process can be deposited into the body and their toxic effects observed in several target organs [7]–[9]. Studies conducted in vitro and using animal models suggested male reproductive toxicity by mercury [10]–[12]. Thus, mercury, at high levels of exposure, affects germ cell development and promotes germ cell injury [13], alters reproductive behavior [11] and decreases sperm count and motility [14], [15]. However, in humans results are conflicting and contradictory [16]; thus, it has been reported reproductive toxic effects of mercury among occupationally exposed workers such as sperm damage and hormonal imbalance [12], [17], [18] as well as an association between semen mercury concentrations and sperm abnormalities in subfertile men [18]; however, other studies did not observe any relationship between blood mercury levels and biomarkers of male reproductive health [19], [20].

It has been proposed that heavy metals can interrupt the normal function of male reproductive system: a) at any level of hypothalamic – pituitary - testicular axis; b) directly, at reproductive organs, or c) by altering post- testicular events such as sperm capacitation, motility and function [21]. Although heavy metals are known as endocrine disruptors, the endocrine implications in the mercury toxicity remained unexplored [21]. Moreover, changes in the hormonal levels caused by mercury treatment at high doses were reported, with decreased testosterone, luteinizing hormone (LH) and follicle stimulating hormone (FSH) levels, suggesting an interference with neuroendocrine system [13], [22].

Involvement of oxidative mechanisms in the toxic manifestation induced by mercury in the reproductive system has been recently investigated, but it is still quite insufficient [14], [23]. Mercury toxicity in several organs has been related with increased reactive oxygen species (ROS) and inactivated antioxidant enzymes necessary for cellular protection against ROS attack [8],[24].

While a good deal of research has been conducted on the toxic effects of mercury, little is known about the underlying mechanisms pathogenesis of male reproductive dysfunction induced by mercury. Recently, we observed decreases on sperm quality after 30 days of mercury exposure and its association with oxidative stress [25]; however, the effects and mechanisms involved after a long-term exposure, which best mimetize human exposure to this metal, are not explored. Therefore, the purpose of this study was to assess the effects of prolonged mercury exposure at low levels on sperm parameters (count, morphology and motility), hormonal levels as well as on biomarkers of oxidative stress on male reproductive system of rats.

## Materials and Methods

### Animals

Three-month-old male Wistar rats (280–320 g) were obtained from the Central Animal Laboratory of the Federal University of Santa Maria, Rio Grande do Sul, Brazil. During treatment, rats were housed at a constant room temperature, humidity, and light cycle (12∶12 h light-dark), free access to tap water and fed with standard chow ad libitum. All experiments were conducted in compliance with the guidelines for biomedical research stated by the Brazilian Societies of Experimental Biology and approved by the Ethics Committee on Animal Use Experimentation of the Federal University of Pampa, CEUA, Uruguaiana, Rio Grande do Sul, Brazil (Process Number: 036/2012).

Rats were divided into two groups (n = 10 each) and treated for 60 days as follows: a) Control (saline solution, i.m.); b) Mercury (HgCl_2_ - mercury chloride (1^st^ dose 4.6 µg/kg, subsequent doses 0.07 µg/kg/day, i.m., to cover daily loss, using the model described by Wiggers *et al*. [26], with an extended exposure for 60 days). At the end of treatment, animals were euthanized by decapitation and the weights of prostate, seminal vesicle (empty, without coagulation gland), testis, epididymis and vas deferens (absolute and relative to body weights) were determined. Prostate, left testis and epididymis as well as right vas deferens were used for further analysis of oxidative stress; the right testis, epididymis and left vas deferens were used for sperm parameter analysis.

HgCl_2_ was purchased from Sigma-Aldrich (St Louis, MO, USA). Salts and reagents were of analytical grade obtained from Sigma and Merk (Darmstadt, Germany).

### Sperm motility

Sperm were removed from the vas deferens by internal rising with 1 mL of Human Tubular Fluid (DMPBS-Nutricell-SP-Brazil) pre-warmed to 34°C. Then, a 10 µL aliquot was transferred to a histological slide. Under a light microscope (20X magnification, Binocular, Olympus CX31, Tokyo, Japan), 100 spermatozoa were analyzed and classified as type A: motile with progressive movement, type B: motile without progressive movement and type C: immotile. Sperm motility was expressed as % of total sperm [25].

### Sperm morphology

Sperm were obtained from the vas deferens and stored with 1 mL of saline - formol until the analysis. For the analysis, smears were prepared on histological slides and 200 spermatozoa per animal were evaluated under 400X magnification (Binocular, Olympus CX31). Morphological abnormalities were classified into head (amorphous, banana and detached head) and tail morphology (bent and broken tail), according to Filler [27].

### Daily sperm production per testis, sperm number and transit time in epididymis

Homogenization-resistant testicular spermatids (stage 19 of spermiogenesis) and sperm in the caput/corpus epididymis and cauda epididymis were counted as described by Robb et al. [28]. To calculate daily sperm production, the number of spermatids at stage 19 was divided by 6.1, which is the number of days these spermatids are present in the seminiferous epithelium. The sperm transit time through the epididymis was determined by dividing the number of sperm in each portion by the daily sperm production [28].

### Biochemical assays

The reproductive organs (testis, epididymis, prostate and vas deferens) were immediately excised after euthanasia, washed on ice-cold saline and rapidly homogenized in 50 mM Tris–HCl, pH 7.5 (1/5, w/v), centrifuged at 2400×g for 15 min at 4°C and the resulting supernatant (S1) fraction was frozen at −80°C for further measurements of oxidative stress parameters.

### Lipid peroxidation

The levels of lipid peroxidation were measured as malondialdehyde (MDA) using a colorimetric method, as previously described by Ohkawa *et al*. [29], with modifications. An aliquot of each tissue was incubated with thiobarbituric acid 0.8% (TBA), phosphoric acid buffer 1% (H_3_PO_4_), and sodium dodecil sulphate 0.8% (SDS) at 100°C for 60 min. The color reaction was measured at 532 nm against blanks (Spectrophotometer Femto 600 S, FEMTO, São Paulo, Brazil). The results were expressed as nanomoles of MDA per gram of protein.

### Determination of non-protein thiols

The non-protein thiols (NPSH) levels were determined by the method of Ellman [30]; for that, homogenates were centrifuged at 4000×g at 4°C for 10 min and the supernatant was mixed (1∶1) with 10% trichloroacetic acid. After centrifugation, the protein pellet was discarded and free -SH groups were determined in the clear supernatant. An aliquot of supernatant was added in potassium phosphate buffer 1 M, pH 7.4, and 5,5′- dithio-bis (2-nitrobenzoic acid) (DTNB) 10 mM. The color reaction was measured spectrophotometrically at 412 nm. The results were expressed as nmol of NPSH per milligram of protein.

### Superoxide dismutase activity

Superoxide dismutase (SOD) activity was spectrophotometrically assayed as described by Misra and Fridovich [31]. This method is based on the capacity of SOD in inhibiting autoxidation of adrenaline to adrenochrome. The color reaction was measured at 480 nm. One unit of enzyme was defined as the amount of enzyme required to inhibit the rate of epinephrine autoxidation by 50% at 26°C. For analysis, tissues were diluted 1∶10 (v/v) and aliquots were added in a glycine buffer 50 mM, pH 10.5. Enzymatic reaction was started by adding epinephrine, and results were expressed as Units (U) per milligram of protein.

### Catalase activity

The catalase activity was spectrophotometrically assayed by the method of Aebi [32], which measures the disappearance of H_2_O_2_ in the presence of tissue homogenates at 240 nm. An aliquot of each tissue was added in potassium phosphate buffer 50 mM, pH 7.0, and the enzymatic reaction was initiated by adding H_2_O_2_. The enzymatic activity was expressed in Units (1 U decomposes 1 µmol H_2_O_2_/min at pH 7 at 25°C) per milligram of protein. Proteins for experiments were measured using bovine serum albumin as a standard.

### Hormonal assay

Blood was collected (between 9:00 and 11:30 AM) and serum was obtained by centrifugation (1236xg for 20 min at 4°C). The concentration of LH and testosterone was determined by the technique of double antibody radioimmunoassay. The hormonal assay was performed using specific kits supplied by the National Institute of Arthritis, Diabetes and Kidney Diseases (NIADDK, USA). All samples were assayed in duplicate and in the same assay to avoid inter-assay errors. The intra-assay error was 3.4%.

### Statistical analysis

Data are expressed as mean ± SEM of the number of animals used in each experiment. Statistical analysis was performed using Student's t test and Mann-Whitney test. Values of *p*<0.05 were considered statistically significant. Graphpad prism 5 software was used for statistical analysis and for plotting graphs.

## Results

Long-term HgCl_2_ exposure at low levels did not change the body weight as well as the absolute and relative organ weights (Table 1).

**Table 1:** Effect of a chronic treatment for 60 days at low concentrations of HgCl_2_ on body weight (g), absolute (g or mg) and relative (g/100 g or mg/100 g) weights of reproductive organs of male rats.

Parameters	Experimental groups	
	Control (n = 10)	HgCl_2_ (n = 10)
Initial body weight (g)	294.9±2.2	301.7±2.9
Final body weight (g)	384.8±18.7	400.7±9.4
Testis (g)	1.8±0.03	1.7±0.04
Testis (g/100 g)	0.4±0.02	0.4±0.01
Epididymis (mg)	671.9±16.1	651.7±17.4
Epididymis (mg/100 g)	177.1±6.3	163.0±3.8
Ventral prostate (mg)	546.0±37.2	507.2±22.4
Ventral prostate (mg/100 g)	142.7±7.7	127.0±5.6
Full seminal vesicle (g)	1.4±0.05	1.5±0.05
Full seminal vesicle (g/100 g)	0.4±0.02	0.4±0.01
Empty seminal vesicle (g)	0.4±0.02	0.4±0.02
Empty seminal vesicle (g/100 g)	0.1±0.01	0.1±0.01
Vesicular secretion (g)	0.9±0.04	1.0±0.04
Vas deferens (mg)	108.4±3.6	107.5±5.7
Vas deferens (mg/100 g)	28.5±1.1	26.8±1.4

Data are expressed as means ± SEM. The relative organ weight was calculated by use of the formula: organ weight/body weight ×100. Units: g: gram, mg: milligram.

### Sperm parameters

Mercuric chloride administration for 60 days resulted in a significant decrease in sperm count in testis and epididymis (caput/corpus and cauda). Furthermore, there was a reduction in daily sperm production per testis in HgCl_2_ treated rats (Table 2).

**Table 2:** Effect of a chronic treatment for 60 days at low concentrations of HgCl_2_ on sperm counts in testis and epididymis of rats.

Parameters	Experimental groups	
*Sperm count*	Control (n = 10)	HgCl_2_ (n = 10)
*Testis*		
Sperm number (x10^6^)	184.1±7.4	138.6±5.2**
Sperm number (x10^6^/g)	101.6±5.1	81.5±3.1**
DSP (x10^6^/testis/day)	29.4±1.3	22.7±0.8**
DSPr (x10^6^/testis/day/g)	16.6±0.8	13.3±0.5**
*Epididymis*		
*Caput/Corpus*		
Sperm number (x10^6^)	109.0±2.6	90.3±2.2**
Sperm number (x10^6^/g)	295.4±7.8	249.9±7.2**
Sperm transit time (days)	3.6±0.1	4.1±0.2
*Cauda*		
Sperm number (x10^6^)	190.0±13.4	148.8±6.7*
Sperm number (x10^6^/g)	713.3±37.0	640.5±15.1
Sperm transit time (days)	6.5±0.4	6.5±0.2

DSP: daily sperm production; DSPr: daily sperm production relative to testis weight. Units: g: gram, mg: milligram. Data are expressed as mean ± SEM. * *p*<0.05, ** *p*<0.01 (Student's t-test).

Sperm morphology analysis revealed a significant increase in sperm abnormalities in rats treated for 60 days with mercury when compared with the control group. Within head phenotypes, more banana head as well as total head abnormalities were observed; concerning tail morphology, there were more bent tail and total tail abnormalities (Table 3).

**Table 3:** Effect of a chronic treatment for 60 days at low concentrations of HgCl_2_ on sperm morphology of rats.

Parameters	Experimental groups	
	Control (n = 10)	HgCl_2_ (n = 10)
*Normal*	95.5 (92.5–96.7)	86.7 (80.1–90.1)**
*Head Abnormalities*		
Amorphous	1.5 (0.6–2.8)	2.7 (1.5–7.5)
Banana Head	0.2 (0.0–0.5)	4.5 (1.7–7.1) **
Detached Head	2.0 (0.5–2.8)	1.7 (1.0–3.8)
Total of Head Abnormalities	3.7 (3.5–5.1)	11.0 (6.7–13.6)**
*Tail Abnormalities*		
Bent Tail	0.5 (0.0–1.0)	2.2 (1.3–4.6)**
Broken Tail	0.5 (0.0–1.2)	0.7 (0.0–1.5)
Total of Tail Abnormalities	1.2 (0.2–1.5)	3.2 (1.8–5.1)**

Data are in percentage and expressed as median (Q_1_–Q_3_). **p<0.01 (Mann – Whitney).

**Figure 1:**
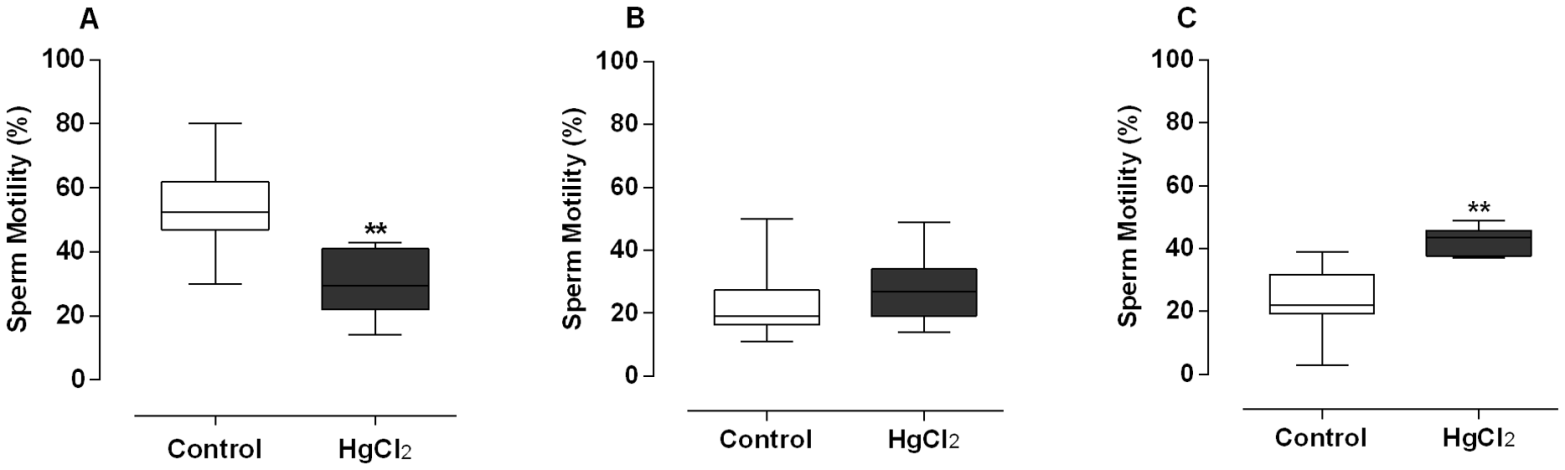
Effect of treatment for 60 days at low concentrations of HgCl_2_ on sperm motility. Type A: motile with progressive movement (A), type B: motile without progressive movement (B) and type C: immotile (C). Data are expressed as median (Q1–Q3), (n = 10). **p<0.01 (Mann – Whitney).

Regarding the sperm motility, we observed decreased type A sperm (motile with progressive movement) (Figure 1A) accompanied by increases in type C sperm (immotile) in HgCl_2_-treated rats (Figure 1C), without differences in type B sperm (motile without progressive movement) (Figure 1B).

### Biochemical assays

**Figure 2:**
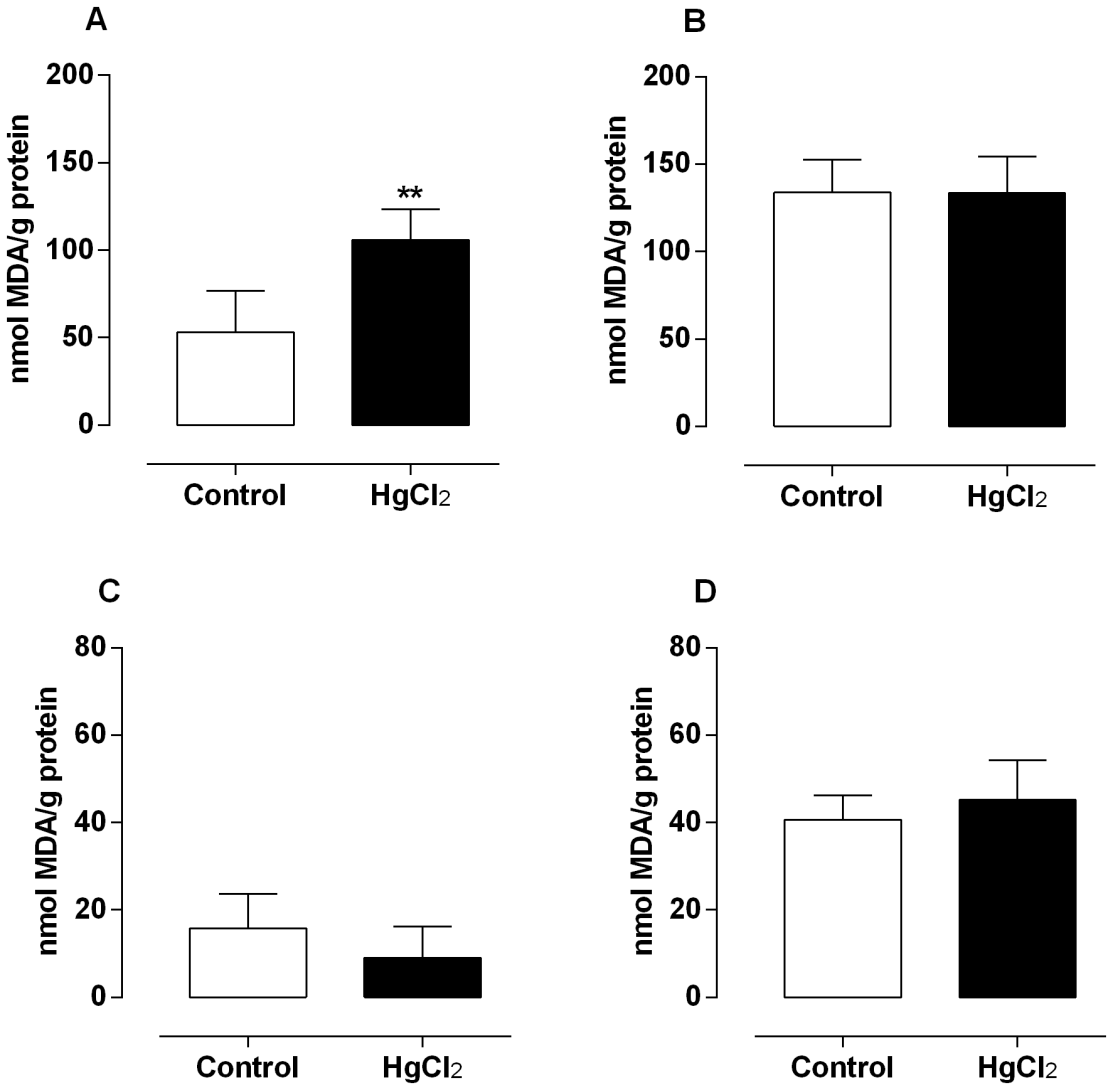
Effect of treatment for 60 days at low concentrations of HgCl_2_ on lipid peroxidation. Values of TBARS on testis (A), epididymis (B), prostate (C) and vas deferens (D). Data are expressed as mean ± SEM (n = 6). ** *p*<0.01 (Student's t-test).

In rats exposed to mercury for 60 days a significant increase on MDA levels was observed in testis (Figure 2A), whereas non-significant changes were observed in epididymis, prostate and vas deferens (Figure 2B, C, D).

**Figure 3:**
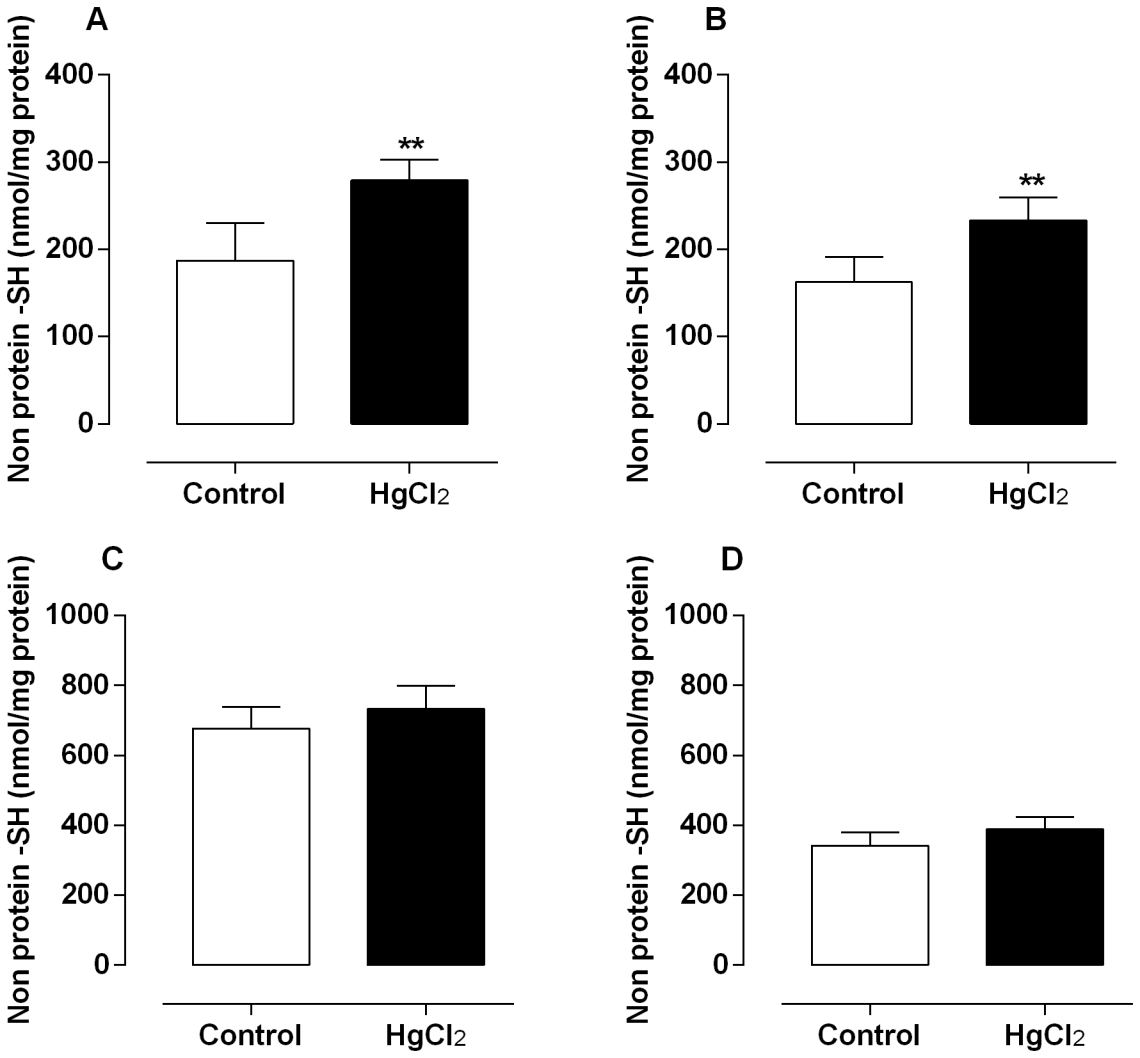
Effect of treatment for 60 days at low concentrations of HgCl_2_ on non-protein thiol content. Values of non-protein thiol groups on testis (A), epididymis (B), prostate (C) and vas deferens (D). Data are expressed as mean ± SEM (n = 6). ** *p*<0.01 (Student's t-test).

With respect to NPSH levels, mercury treatment increased the testicular and epididymal SH levels (Figure 3A, B), while those in prostate and vas deferens were not statistically different between groups (Figure 3C, D).

**Figure 4:**
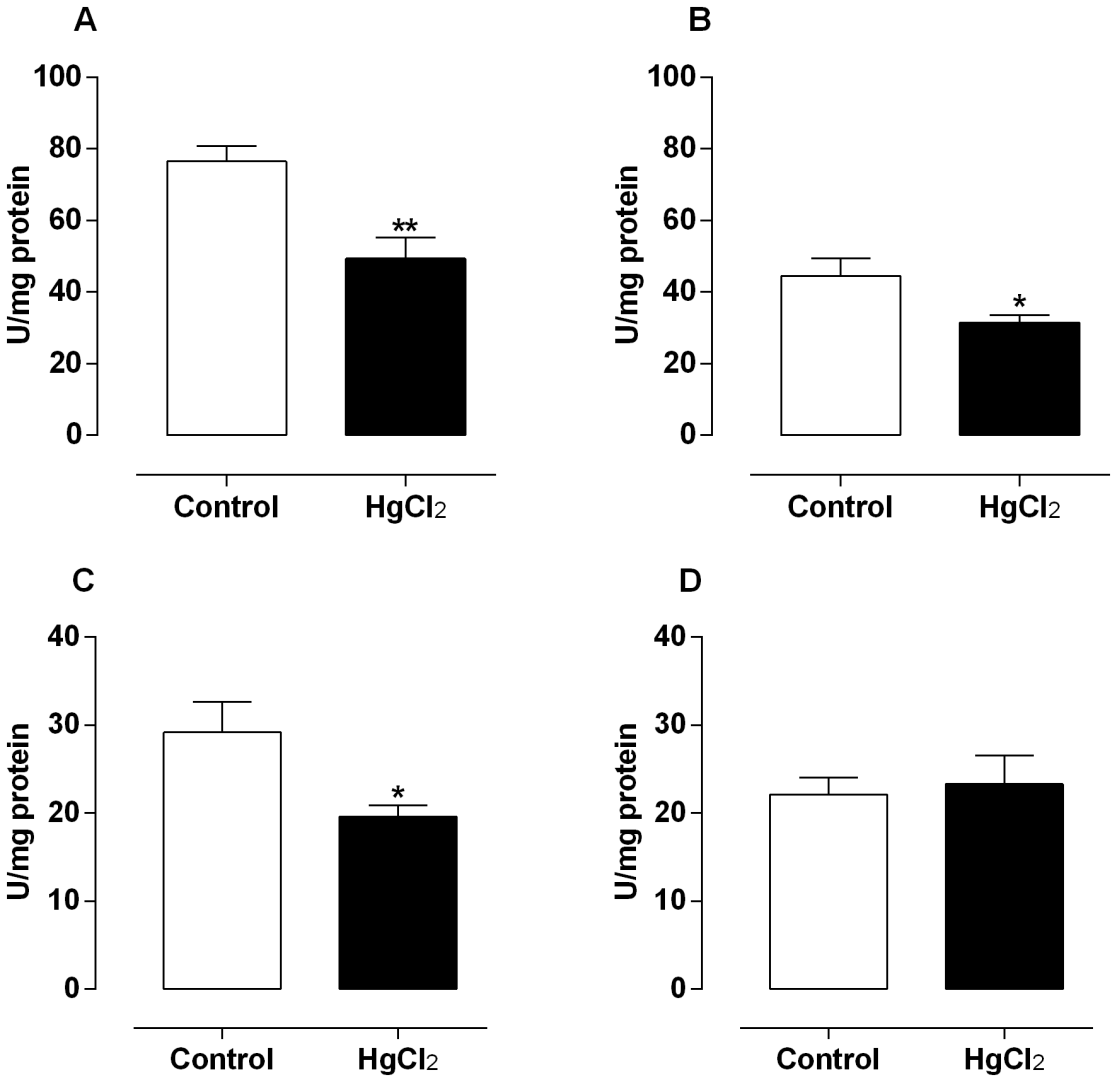
Effect of treatment for 60 days at low concentrations of HgCl_2_ on superoxide dismutase activity. Values of superoxide dismutase activity on testis (A), epididymis (B), prostate (C) and vas deferens (D). Data are expressed as mean ± SEM (n = 6). ** *p*<0.01, *p<0.05 (Student's t-test).

**Figure 5:**
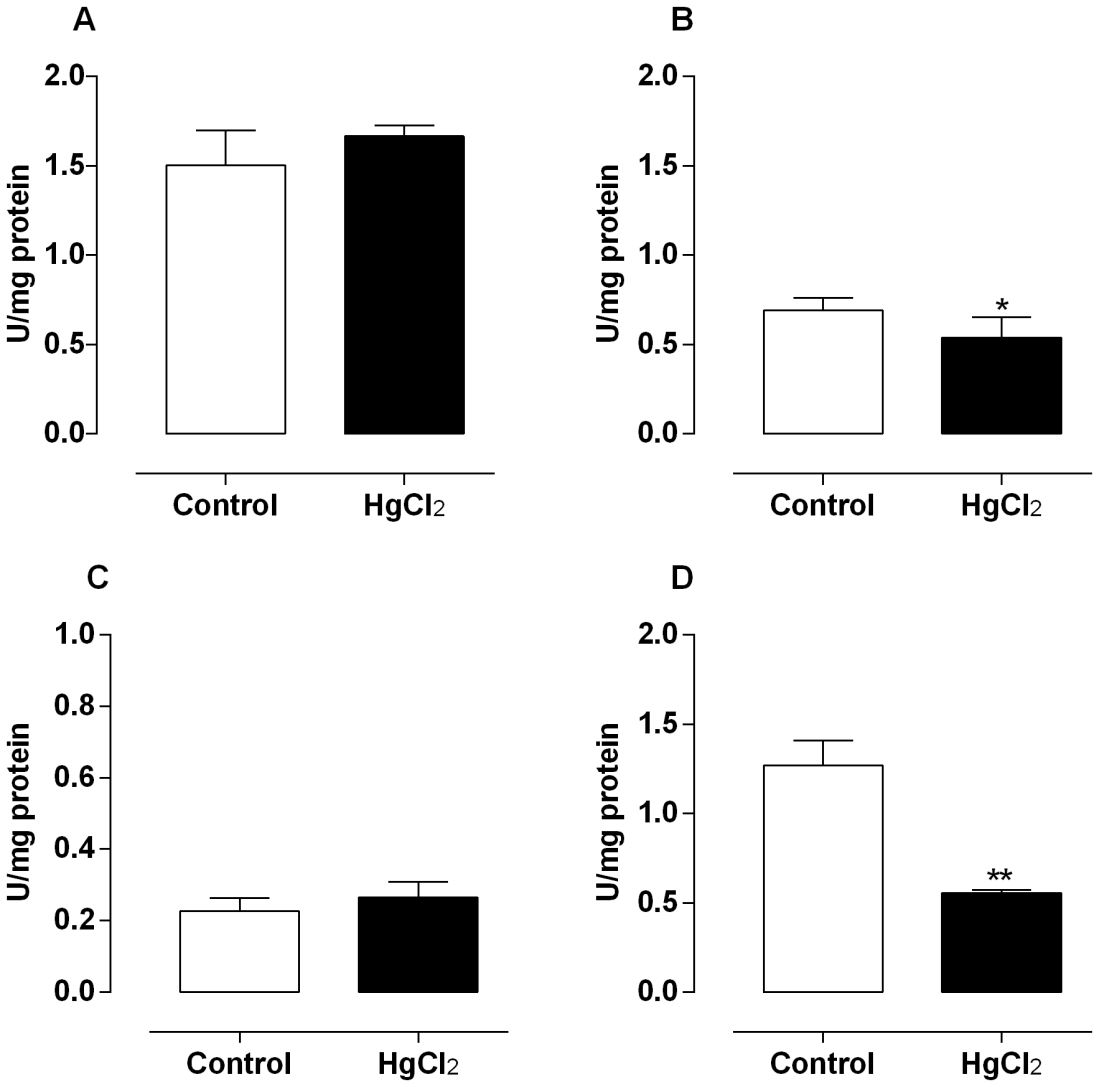
Effect of treatment for 60 days at low concentrations of HgCl_2_ on catalase activity. Values of catalase activity on testis (A), epididymis (B), prostate (C) and vas deferens (D). Data are expressed as mean ± SEM (n = 6). ** *p*<0.01, *p<0.05 (Student's t-test).

The mercury exposure for 60 days decreased the enzymatic activity of SOD in testis, epididymis and prostate (Figure 4A, B, C), without change in rats vas deferens (Figure 4D). Moreover, the catalase activity after mercury treatment slightly decreased in epididymis and in a great extent in vas deferens of rats (Figure 5B, D); however, in testis and prostate there were not differences in the activity of this enzyme (Figure 5A, C).

### Hormonal assay

**Figure 6:**
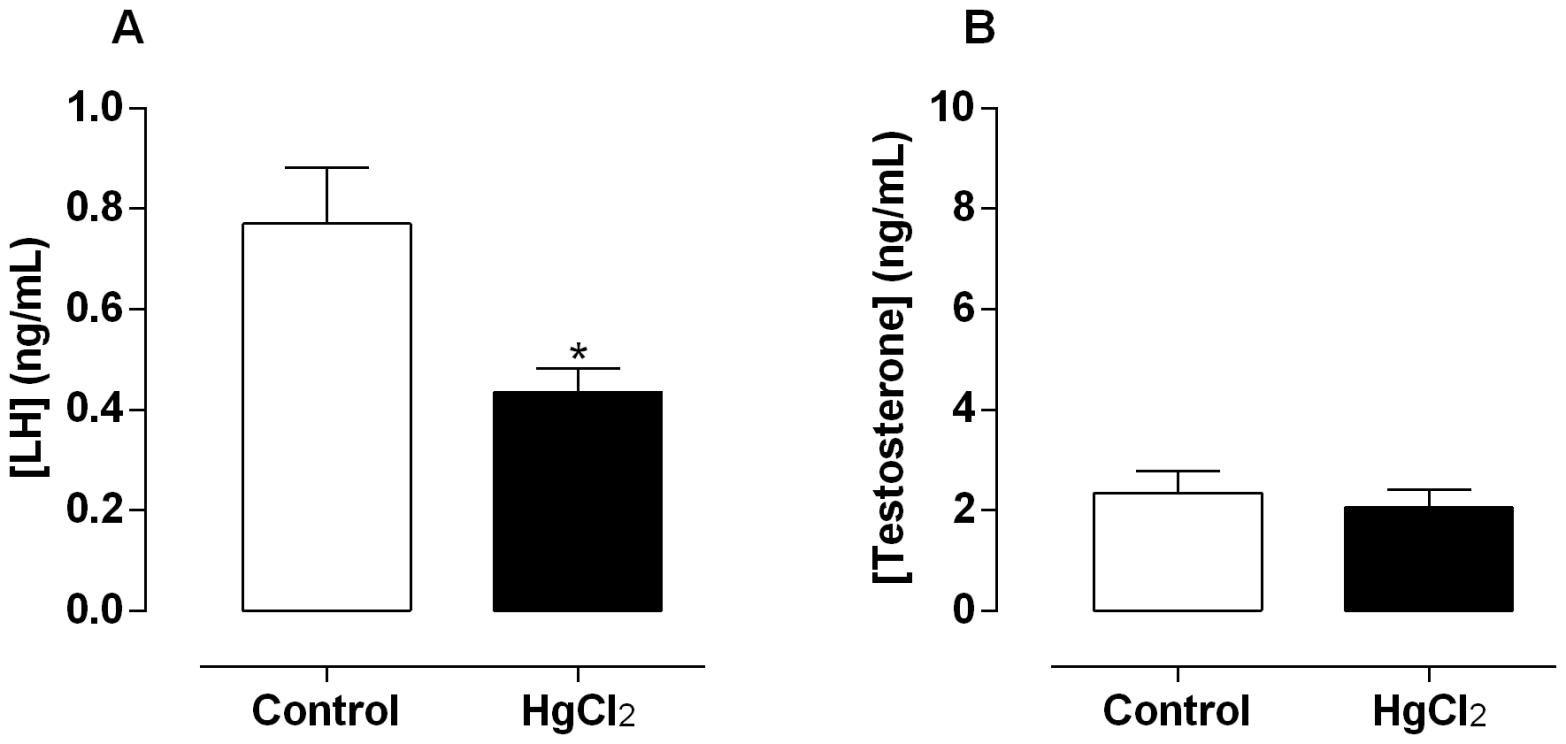
Effect of treatment for 60 days at low concentrations of HgCl_2_ on serum hormonal levels. (A) Luteinizing hormone (LH) and testosterone levels (B). Data are expressed as mean ± SEM (n = 10). *p<0.05 (Student's t-test).

There was a significant reduction in serum levels of LH in the mercury treated rats when compared with the control group (Figure 6A), with no differences in testosterone levels after mercury exposition (Figure 6B).

## Discussion

In a previous report, we have shown that 30 days exposure to HgCl_2_ induces male reproductive dysfunction associated to increased oxidative stress. The present study goes further in this field showing that when mercury exposure is prolonged for 60 days, this metal cumulatively affects the male reproductive system. The current study shows that marked changes after 30 days of mercury administration are maintained, although the defense mechanisms are differently affected after a prolonged mercury exposition. Long-term HgCl_2_ administration impairs sperm function, as observed by suppression on sperm production and count reduction following by motility and morphological abnormalities mainly banana head and bent tail; and also caused hormonal disparity with decreased LH levels. These effects seem to be related with increased oxidative stress. Namely, this investigation demonstrates a fail from the adaptative response of SOD activity as well as an apparently improve from lipid damage when compared with 30 days of exposure.

Mercury is suspected to have negative impact on male fertility, although human studies are few and contradictory [12], [16], [18]. The United States Environmental Protection Agency (EPA) recommends a maximum blood concentration of mercury of 5.8 ng/ml [5], [6]; however the human exposure to mercury is still increasing and sometimes are higher than allowed [4], [33]. In the present study, we used a model of controlled low-dose administration of HgCl_2_ for 60 days which was previously established in rats [26] and attains a blood mercury concentration of approximately 8 ng/ml, similar to human exposure levels and near to those considered safe by the EPA [19], [34].

Animal studies with 30, 60 and 90 days of mercury exposure at high levels had showed decreased testosterone, LH, FSH and prolactin levels [13], [22], [23]. Recently, within the US population, it was discovered a significant inverse relationship between chronic inorganic mercury and LH levels [3]. Our study raises a question about the association between chronic mercury exposure at a dose environmental relevant and LH as a causal mechanism for mercury associated diseases; the decreased serum LH levels highlights mercury as an endocrine disruptor. Endocrine disruptors are an exogenous agent that either mimic or block the effects of hormones at the target receptor or by directly stimulating or inhibiting production of hormones [21], being the complex hypothalamus–pituitary–gonad (HPG) axis within the domain of endocrine disruption [35].

The relationship between chronic mercury exposure and LH was first postulated considering that pituitary gland is one of the target for mercury deposition within the brain [36]. Even that, relatively little mercury in inorganic form crosses the blood brain barrier; however, inorganic mercury can be formed as a metabolite of other forms of mercury in the brain where it can remain for years [36], [37]. Moreover, the bran effect-response to mercury seems to be dependent on reaching a critical threshold concentration/deposition after prolonged exposure, since we did not find LH reduction after 30-days exposure. This hypothesis is supported by a recent publication of our group in which we observed that only exposure for 60 days to HgCl_2_ in adulthood can cause deleterious effects on cognitive function and memory formation [38]. However, further experiments are still necessary to determine the time necessary for the effects we observed.

In the present study we did not find changes in either body weight or reproductive organs weight, similar to that previously found after 30 days exposure [25]. The fact that the testosterone levels were maintained supports these results because the reproductive system is directly under control of androgen stimulation [39], [40].

The 60-day HgCl_2_ exposure decreased sperm motility, daily sperm production and sperm quantity in testis and epididymis; in addition, we also observed a significantly increase in sperm abnormalities in exposed-animals. These impairments are similar to that found in our previous study in terms of percentage [25], except for sperm motility, in which a prolonged mercury exposure seems to be more deleterious to sperm movement. Our results are in agreement with other authors using HgCl_2_ 3 times a week at high doses for 60 days [13]. Similarly, inorganic mercury at 0.25–1.00 mg/kg/day (21 days) produced adverse effects on mice reproductive performance with fertility and sperm survival reduction [11].

Other heavy metals such as lead, cadmium and arsenic are also known to induce reproductive toxicity, including abnormalities in sperm count, motility, morphology, testosterone production and spermatogenesis [21], [41], [42]. One of the target organs of heavy metals is testis and many authors have proposed that oxidative stress could be partially responsible to induce their toxicity [43], [21].

In this sense, HgCl_2_ has been reported to be one of the most pro–oxidant forms that induce oxidative stress in several organs [8], [9]. Recently, this ability has been shown on male reproductive organs at high mercury levels [13], [14] as well as at low levels in our previous study after 30 days of HgCl_2_ exposition [25]. The current investigation suggests that oxidative stress might be one of the mechanisms explaining mercury reproductive toxicity such as hypospermatogenesis, as demonstrated by the lipid peroxidation enhancement in testis as well as the reduction in antioxidant enzymes activities in reproductive organs. The apparent discrepancies between increased lipid peroxidation in testis while not differences in other evaluated organs were found, even with reduction in antioxidant status, are expected in metal exposure due to differences in metal accumulation and tissue response to insults according to specific characteristic of this tissue [44].

ROS are important mediators of normal sperm function, but excessive production of ROS results in lipid peroxidation and membrane damage, inactivation of glycolytic enzymes, damage to the acrosomal membranes and DNA oxidation [45], [46]. Moreover, mammalian spermatozoa are rich in polyunsaturated fatty acids (PUFAs) which are very susceptible to ROS attack. As a consequence of lipid peroxidation, PUFAs undergo degradation by a chain reaction leading to production of cytosolic aldehydes including MDA as well as loss of structural integrity required for sperm viability and motility [45], [46]. Therefore, the suppression of spermatogenesis and sperm impairment found would be attributed to peroxidation of PUFAs in the sperm membrane.

Mercury compounds have high affinity to thiol (-SH) groups of biomolecules; depletion of intracellular thiols has been reported as a trigger for production of ROS by mercury [47]. The enhanced non protein -SH levels in testis and epididymis found after 60 days exposure could constitute an adaptive response to oxidative stress, since at 30-day of HgCl_2_ exposure depletion of intracellular thiols in testis and epididymis of rats was observed [25].

Cells have several mechanisms to protect themselves from the toxic effect of ROS [48]. It is known the protective effect of the antioxidant enzymes SOD, CAT and GPx against toxicity induced by mercury [9], [13], [47]. Long-term exposure to mercury decreased SOD activity in testis, epididymis and prostate as well as catalase activity in epididymis and vas deferens; these reductions can raise the oxidative damage due to accumulation of superoxide radicals and hydrogen peroxide [14], [23]. Catalase activity was also reduced after 30 days treatment, while SOD activity was increased in all reproductive organs, probably as a compensatory mechanism [25]; therefore, the lack of enzyme activity here found suggests a failure of antioxidant defense SOD after a prolonged mercury exposure. The reduction of SOD and CAT activities could be due either to a reduction on these enzymes expression, to a direct effect of ROS, or to a direct inhibition from mercury [48].

The present study raises concern about the toxicity of long-term HgCl_2_ exposure, since another 30 days of mercury administration at environmental relevant levels was more deleterious and insufficient to a compensatory positive reaction from endogenous antioxidants at male reproductive system. Corroborating, Sharma et al. [49] after mercury administration of 1.25 mg/kg daily for 30 days observed only a partial recover after withdrawal of HgCl_2_ for 45 days. Thus, further experiments are necessary to determine the time for recovery of the reproductive system effects.

## Conclusions

Our results demonstrate that 60-day chronic exposure to low concentrations of HgCl_2_ impairs sperm quality and promotes hormonal imbalance with decreased LH levels. The raised oxidative stress seems to be a potential mechanism involved in male reproductive toxicity by mercury.
